# Validity of the Strengths and Difficulties Questionnaire for Screening and Diagnosis in Western Australian Adolescents

**DOI:** 10.3390/diagnostics14212433

**Published:** 2024-10-30

**Authors:** Carolyn Maxwell, Elaine Chapman, Stephen Houghton

**Affiliations:** Graduate School of Education, The University of Western Australia, 35 Stirling Highway, Perth 6009, Australia; carolyn.maxwell@uwa.edu.au (C.M.); stephen.houghton@uwa.edu.au (S.H.)

**Keywords:** strengths and difficulties questionnaire, SDQ, validity, instrument validation, ADHD

## Abstract

Background/Objectives: The Strengths and Difficulties Questionnaire (SDQ) is a widely used 25-item screening and diagnostic tool for behavioral and emotional problems in young people. Despite its popularity, evaluations of the SDQ’s factor structure in adolescent populations have produced disparate results, and its relationships with theoretically related variables are rarely evaluated. In the present study, these two elements of validity were evaluated based on a large sample of Western Australian adolescents. Methods: Participants were 1489 adolescents, *n* = 623 males with a mean age of 13.79 years (*SD* = 1.61) and *n* = 866 females, with a mean age of 14.29 years (*SD* = 1.51). Participants completed the SDQ alongside measures of loneliness, sense of belonging, depression, bullying, and diagnostic status to evaluate its internal structure and correlations with theoretically related variables. Results: Confirmatory factor analyses supported the internal structure of the SDQ both for males and for females. Relationships between the SDQ subscale scores and those from theoretically related variables were also aligned with the instrument’s underpinning framework. Conclusions: Despite the somewhat disparate results of previous studies, overall, this study supported the validity of the SDQ for use in the Western Australian context.

## 1. Introduction

While the prevalence of mental disorders has remained fairly constant, the reported incidence of mental health problems during the adolescent years is increasing worldwide. Recent studies have indicated that mental health difficulties are now reported in around 20% of adolescents [1,2]. Poor mental health in young people is an important predictor of adverse life circumstances in adulthood. For example, poor mental health in adolescence has been linked to societal disadvantage, psychological problems, and an increased likelihood of dependence on welfare and unemployment throughout the adult years [3,4,5]. Thus, poor mental health in young people has consequences that extend well beyond the period of formal schooling and should be seen as a growing global crisis for all citizens [6]. Early identification, however, can improve long-term prognoses for affected individuals [7,8].

Schools provide convenient settings in which early interventions for mental health problems may take place. Most young people are enrolled in formal schooling, which increases the breadth of the impact that such programs can have. In Australia, significant investments from the Federal Government have been made over the last 20 years to increase awareness of mental health problems and mental health service support for young people of school age. Despite such investments, our ability to identify and prevent youth mental health problems has seemingly not improved. This may be attributable in part to the fact that wide-scale screening for mental health difficulties remains rare in Australian schools, which may, in turn, reflect a need for screening systems that are better suited for use in such contexts.

Graybill et al. [9] argued that early identification systems are important for ensuring that those with mental health difficulties are referred appropriately to receive timely support. Various authors have noted the need for psychometrically sound instruments that can be used in school settings to assist with the timely identification of mental health difficulties. Hoofs et al. [10] argued that such instruments are crucial because these enable prompt medical interventions that enhance prognoses for mental health. He et al. [11] and de Vries et al. [12] further argued that regular broad-scale mental health screening is key to appropriate targeting of timely support interventions.

### 1.1. Instruments Available for School-Based Screening of Mental Health Problems

While various instruments that assess certain aspects of mental health have been published, many such instruments are relatively narrow in focus. As argued by Rothenberger and Woerner [13], behavioral assessments need to incorporate a diverse range of behavioral difficulties to provide a thorough indication of a young person’s psychiatric status. Failing to obtain such a thorough assessment can hamper treatment planning. Coupled with the narrow focus of many instruments relating to mental health in youth, schools that wish to monitor the mental health of students on a routine basis may find that they need to use a battery of tests to achieve a broad view of their mental health.

A small number of instruments currently available may be suitable for use as broad-based mental health screening tools in terms of their content. For example, the Achenbach System of Empirically Based Assessment (ASEBA), which incorporates the Child Behavior Checklist (CBCL), offers a thorough assessment of functioning (both adaptive and maladaptive). This assessment also has a Youth Self-Report version that could be used in school settings. The ASEBA assesses mental health across a range of factors, including social and thought problems, depression and anxiety, and attentional concerns.

While the ASEBA provides a comprehensive assessment of mental health problems in young people, it is a commercial instrument. In addition to being reticent about investing time in assessing for regular mental health screening, many schools are not in a position to purchase access to such instruments on a broader scale [9]. The lengthiness of these tools can also make them unsuitable for use in many cases, particularly against the backdrop of an already crowded curriculum that many schools currently confront. Similar considerations apply to a number of tools that might otherwise be suitable for use in schools, including the BASC–3 Behavioral and Emotional Screening System (BESS), which explores behavioral and emotional strengths and weaknesses, and the Pediatric Symptom Checklist (PSC), which explores behavioral and emotional health problems. Both instruments are suitable for use by different informants (students, teachers, or parents).

Another group of instruments, also commercially available, rely primarily on teachers to provide ratings of student behavior. This group includes the Systematic Screening for Behavior Disorders (SSBD), which explores externalizing and internalizing behavior and is suitable for students from the ages of 3 to 15; the Social, Academic, and Emotional Behavior Risk Screener (SAEBRS), which explores the social, academic, and emotional behavior of students, and is suitable for those aged 5 to 18 years; and the Student Risk Screening Scale (SRSS), which explores externalizing and internalizing behavior and is suitable for those aged 5 to 18. Since all these instruments rely upon teacher ratings of behavior, they may be unsuitable for regular monitoring of student mental health on a broad-scale basis, given the prohibitive time commitments required for their completion.

### 1.2. The Strengths and Difficulties Questionnaire

The Strengths and Difficulties Questionnaire (SDQ) by Goodman [14] is a 25-item, publicly available, and widely used screening tool for mental health problems in children and adolescents. The content of the SDQ aligns with diagnostic categories in the Diagnostic and Statistical Manual of Mental Disorders, Fourth Edition (DSM-IV) [15] and with those in the current fifth edition (DSM-5) [16]. The SDQ is suitable for use with young people aged between 2 and 17 years and is available in six versions, including both the Parent and Youth Self-Report versions (see Table 1).

All versions of the SDQ ask about 25 negative or positive attributes of the young person in question, which are clustered into five groups or subscales of five items each: Emotional Symptoms; Conduct Problems; Hyperactivity/Inattention; Peer Relationship Problems; and Prosocial Behavior. While the theoretical structure of the SDQ is generally posited to comprise these five subscales, there is also a proposed three-dimension structure in which ‘internalizing problems’ (emotional and peer symptoms, 10 items), ‘externalizing problems’ (conduct and hyperactivity symptoms, 10 items), and prosocial subscales have been grouped [14]. This structure has, however, attracted limited support within the literature. In using the original five-factor SDQ, item scores from the first four subscales (Emotional Symptoms; Conduct Problems; Hyperactivity/Inattention; Peer Relationship Problems) can be summed to produce an overall difficulties score.

Parents and teachers can complete questionnaires based on these 25 items for young people aged between 4 and 17 years [14]. A slightly modified version of the same 25 items is completed by parents and teachers of students aged from 2 to 4 years. Questionnaires for self-completion by adolescents in the Youth Self-Report version of the SDQ ask about the same 25 characteristics, though the wording has been adjusted to a more appropriate level for young persons. Houghton et al. [17] have found that accurate depictions of subjective dispositions can be obtained through the use of self-report instruments relating to internal mental states.

The SDQ may be a viable candidate for use as a broad-based screening tool for several reasons. First, its brevity makes it a more practical option than alternative measures [18,19,20]. Second, the SDQ has been demonstrated to have comparable reliability and validity to longer instruments [19]. Third, the SDQ’s strong theoretical basis and its focus on strengths as well as weaknesses make it particularly suitable for use in school-aged populations [21].

### 1.3. Validity of the Strengths and Difficulties Questionnaire

While screening tools are designed to assess individuals on a broad-scale basis, all assessment tools will have some washback effects and, thus, the stakes attached to the use of these tools are significant. False positive results, for example, will call upon schools to waste resources in following up with students who have been identified to be at risk and may stigmatize some students needlessly. It is thus essential that such tools not only meet criteria in terms of practicality, cost, and conceptual soundness but also that they are demonstrated empirically to align with the constructs intended.

The 2014 Standards for Educational and Psychological Testing [22] define validity as “the degree to which evidence and theory support the interpretations of test scores for proposed uses of tests” (p. 11). In this framework, the validity of the scores generated by an instrument should be evaluated in terms of evidence regarding (i) the test content (e.g., the extent to which the content domain is represented in the test items); (ii) the response processes associated with the instrument (e.g., what individuals need to do to respond to the test items); (iii) the internal structure (e.g., whether the factorial structure aligns with the theoretical basis of the instrument); (iv) relations to other variables (e.g., correlations between the instrument scores and those from instruments that test theoretically related constructs); and (v) usage consequences (e.g., the extent to which the instrument produces identifiable positive effects for individuals).

Of these validity criteria, consequential validity is the most controversial, with many researchers arguing that this notion conflates too many ideas underneath the banner of validity [23,24]. As a result, few evaluations of validity include a consideration of this factor. With respect to the SDQ, its acceptance amongst a wide group of professionals over an extended period provides some evidence to support its content validity. Furthermore, the fact that the SDQ has been widely used with no reported problems in students’ interpretations of the questions [25,26,27] provides some support for its validity in terms of response processes (i.e., the processes associated with the use of the instrument in relevant groups).

There is less compelling evidence in the literature, however, with respect to the SDQ’s internal structure and correlations with theoretically related variables. While various studies have been published in which the internal structure of the SDQ has been evaluated, these evaluations have produced disparate results. Table 2 summarizes the results of studies in the last 23 years that evaluated the internal structure of the SDQ Parent, Teacher, and Youth Self-Report versions across different contexts within this period. As indicated, while some studies [19] obtained results that supported the original five-factor structure of the SDQ, other studies [6] found that correlated error terms needed to be used to achieve adequate fit. Other studies indicated that the proposed factor structure was not tenable regardless of the models tested [25], or that a sound fit was only achieved with respect to specific subscales [28]. Thus, while some previous studies on the internal structure of the Youth Self-Report version of the SDQ have suggested that the original five-factor structure is tenable in some contexts, others have suggested a poor fit of the five-factor model to the data. This underscores the importance of evaluating the internal structure of the instrument in different participant groups.

Similarly, few studies have been published in which the validity of the SDQ has been evaluated with respect to its correlations with other theoretically related variables. For the SDQ Youth Self-Report version, moderate to strong correlations between conceptually similar SDQ and Youth Self-Report (YSR) scales have been reported in only two previous studies [29,30]. For the SDQ Parent Report version, the present authors could locate only one study focused on adolescents. This latter study indicated that there was a moderate correlation between conceptually similar scales of the SDQ Parent Report and the Youth Self-Report (YSR) instruments [30]. In general, validity studies on the SDQ in adolescent populations remain relatively scarce [31]. The authors of the present study, for example, were unable to locate any large-scale empirical evaluations of the Youth Self-Report version of the SDQ in Western Australia. Given that previous studies on the factor structure of the SDQ have yielded disparate results, the first goal of the present study was to evaluate the internal structure of the SDQ in a large sample of Western Australian adolescents. The second goal was to evaluate correlations between the SDQ scores and those from measures of other, theoretically related variables.

**Table 2:** Previous studies on the factor structure of the SDQ *.

Authors	Sample and Location	Results
Ortuño-Sierra et al. [6]	3012 males and females aged 12 to 17 years from Spain, England, Ireland, Germany, and France	Adequate fit of the five-factor model with the addition of correlated errors and permitting reverse-worded items to cross-load on the Prosocial subscale.
De Vries et al. [12]	3451 males and females, mean age 13.7 years in South Africa	Significant item loadings using CFA obtained for the emotional and prosocial behavior subscales on the five-factor model, but not for all items on other subscales.
Mellor & Stokes [32]	914 males and females aged 7 to 17 years in Australia	SDQ subscales were not unidimensional—eight items failed to load in unidimensional models of their respective subscales.
Ellis et al. [28]	386 males and females aged 15 to 18 years in Northern Ireland	Three of the five original factors (Emotional Problems, Prosocial, and Hyperactivity) were supported, but two separate Conduct factors (Aggressive Conduct and an Antisocial Conduct), as well as a Good Behavior factor, emerged.
Black et al. [25]	30,290 males and females aged 11 to 15 years from England	The proposed factor structure did not fit the data well.
Duinhof et al. [33]	33,233 males and females aged 11 to 15 years from Bulgaria, Germany, Greece, the Netherlands, Poland, Romania, Slovenia	Achieving adequate fit required the removal of five reverse-worded items.
Percy et al. [34]	3753 males and females aged 12 years from Northern Ireland	Although the optimum number of latent factors for the SDQ could not be determined in the EFA, a subsequent CFA provided modest support for the original five-factor solution.
Vugteveen et al. [31]	5015 males and females aged 12 to 17 years from the Netherlands	The original five-factor model did not fit the data well—this was attributed to a potential wording effect.
Thompson et al. [35]	550 3½-year-olds, 591 7-year-olds, and 620 11-year-olds in New Zealand	The proposed five-factor structure was supported and found to be robust across three separate timepoints, though several questions failed to load as predicted in the youngest age group.
Bøe et al. [36]	10,254 males and females aged 16 to 18 years from Norway	Acceptable fit obtained for a modestly modified five-factor model.
Karlsson et al. [21]	5549 males and females aged 15 to 16 years in Sweden	Superior fit of the five-factor versus the three-factor model, though neither fit the data well. Incorporating cross-loadings enhanced model fit.
Van Roy et al. [37]	26,269 males and females aged 10 to 19 years from Norway	The five-factor model fit the data to a satisfactory level; fit further enhanced through incorporation of correlated error terms for two item pairs.
Ruchkin et al. [38]	2892 males and females aged 3 to 18 years in Russia	Adequate fit was obtained for all models tested, but factor loadings and scale reliabilities were low.
Muris et al. [39]	562 males and females with a mean age of 12.3 years from the Netherlands	The five-factor solution yielded adequate fit to the data.
Goodman [18]	3983 11–15-year-olds in Britain	The five-factor structure was supported by the data.
Skoczeń et al. [40]	582 males and females with a mean age of 13.88 years in Poland	Of the five proposed SDQ dimensions, only emotional symptoms and prosocial behavior scales were empirically distinct from a general difficulty factor.

* Relevant translated versions used depending on location of the study.

## 2. Materials and Methods

This study formed part of a broader longitudinal project involving adolescents within Western Australia [17]. In the broader study, data were collected across four separate timepoints: Timepoint 1—November 2018; Timepoint 2—April/May 2019; Timepoint 3—March 2020; and Timepoint 4—July/August 2020. The data used in the present study were drawn from Timepoint 1, given that the numbers for all other timepoints were lower than for this first timepoint.

### 2.1. Participants

There were 1489 participants in all, *n* = 623 males (mean age 13.79 years, *SD* = 1.61) and *n* = 866 females (mean age 14.29 years, *SD* = 1.51). Participants were enrolled across seven randomly selected schools in Perth, Western Australia, which represented a range of socioeconomic status intakes based on the Index of Community Socio-Educational Advantage (ICSEA). ICSEA values have an overall average of 1000 (*SD* = 100). Given that ICSEA values in the present study ranged from 939 to 1191, the schools represented a range of socioeconomic status levels, centered around the overall average.

### 2.2. Instruments

All participants completed the SDQ Youth Self-Report baseline version online using the Qualtrics survey software (https://www.qualtrics.com/). Five additional instruments or question sets were also completed by some or all participants, which permitted an evaluation of the correlations between SDQ scores and those from measures of theoretically related variables.

#### 2.2.1. The Perth A-Loneness Scale (PALs)

Two subscales from the self-report PALs [41], a multidimensional measure of loneliness, were used in the present study. The PALs comprises 24 items rated on a six-point scale (never, score = 0 to always, score = 5). A series of factor analytic studies [41,42,43] have indicated that the PALs includes four correlated factors: friendship-related loneliness (e.g., having reliable and trustworthy friends); isolation (e.g., having few friends or limited access to social support); having a positive attitude to being alone (e.g., positive aspects of being alone); and having a negative attitude to being alone (e.g., negative aspects of being alone). The same studies have also indicated that the instrument has excellent psychometric properties in terms of both validity and reliability. Scores from the first two subscales (friendship-related loneliness and isolation) were used in the present study.

#### 2.2.2. The California Healthy Kids Survey (CHKS)

The School Connectedness subscale from the CHKS was used in the present study to provide a measure of participants’ sense of belongingness—that is, whether a student has a positive sense of being accepted by others, as well as their sense of being valued and included in their schools. The CHKS includes elementary- and secondary-level versions. The four items in the secondary CHKS are modified versions of items that were developed to measure school belongingness by Resnick et al. [44]. An evaluation of the psychometric properties of the CHKS has affirmed the internal structure and consistency of the instrument [45]. The four items from the CHKS used in the present study were (i) “I feel close to people at/from this school”; (ii) “I am happy with/to be at this school”; (iii) “I feel like I am part of this school”; and (iv) “I feel safe in my school”.

#### 2.2.3. Children’s Depression Inventory, Self-Report (Short) Version (CDI-2:SR[S])

The third instrument used to validate the SDQ in the present study was a revised version of the Children’s Depression Inventory (CDI). The CDI-2:SR[S] (CDI-2 forthwith) is designed to identify signs of depression in children and is written at a first-grade reading level. The short form typically takes between 5 and 15 min for children to complete and has been reported to have excellent psychometric properties, with studies indicating that both the full version and the short version provide valid assessments when used as screening tools for depression [46].

#### 2.2.4. Researcher-Constructed Questions on Bullying

Respondents answered a total of eight researcher-constructed questions related to bullying: four about being the victim of bullying and four about being the perpetrator of bullying. The four items within each of these subscales measured different forms of bullying, including physical, verbal, social, and cyber bullying. Total scores for frequency were generated for each of these subscales:

Items 1–4. How often have you been physically/verbally/socially/cyber bullied?

Items 5–8. How often have you taken part in physical/verbal/social/cyber bullying?

#### 2.2.5. Questions on Formal Diagnoses

Respondents also answered a series of questions about whether they had received any formal diagnoses of mental disorders (ADHD, ASD, and/or SLD) previously. We used only the ADHD diagnosis question for the purposes of this study: “Have you ever been diagnosed with ADHD?”. Students ticked a single box in response to this question, choosing between the three options: “Yes”, “No”, or “I don’t know”. These self-reported data were then checked by the school principal and/or school psychologist against official school records. At the same time, a check was made for students who may not have self-reported a formal diagnosis. The final responses were thus verified against official records. Those that remained as “I don’t know” were those in which there was no official record of an ADHD diagnosis with the school, but where such a diagnosis may have been assigned without the school being informed.

### 2.3. Procedures

Ethics approval was obtained from the researchers’ institution (The University of Western Australia, ethics approval number 2023/ET000730), as well as from the Western Australian Department of Education (D18/0207029). Approval was also obtained from the principal of each participating school. Informed consent and verbal assent were obtained from individual participants. Participants completed the surveys online during regular school time and received a unique identification code that was used to ensure that any information provided remained confidential. Each participating principal nominated one teacher who took responsibility for overseeing the implementation of the study in that school. These teachers received detailed instructions from the researchers to ensure that the study was administered in a standardized way across the schools.

## 3. Results

Of the 1489 participants who completed the SDQ, not all completed the additional validation measures. These participants, therefore, were retained for the purposes of the factor analysis but excluded for the purposes of evaluating correlations between the SDQ scores and those from other measures. The results obtained with respect to the two elements of validity evaluated in the study (internal structure and relationships with external variables) are considered separately in the next two sections.

### 3.1. Evidence Related to Internal Structure

To evaluate the internal structure of the SDQ for all participants who completed the SDQ, the data file was split by gender into males (*n* = 623) and females (*n* = 866). Confirmatory factor analysis (CFA) models were then tested on the two separate data files using LISREL V11.0. Preliminary investigations of compliance with CFA assumptions indicated no significant violations of assumptions associated with linearity, factorability, and the case-to-item ratio. While moderate levels of skew and leptokurtosis were observed in a number of the item distributions, transformations performed using PRELIS to normal scores produced no differences in the item scores. As a result, the raw scores were retained for use.

The first model tested for each of the groups was a unidimensional model. The second was based on the alternative three-factor model suggested by Goodman et al. [47], in which all items in the Emotional and Peer subscales were grouped into a single ‘internalizing problems’ factor (10 items); all items in the Conduct and Hyperactivity symptoms were grouped into a single ‘externalizing problems’ factor (10 items); and the Prosocial scale remained the same (5 items). The third model tested was the original proposed five-factor SDQ structure. The change in χ^2^ among the models was used to evaluate whether the model fit statistics for the three models were significantly different.

Fit indices for the three models tested for the male and the female subgroups are shown in Table 3. For males, based on the χ^2^ statistics, the five-factor solution was clearly the best-fitting model. Specifically, while the three-factor solution produced a significantly better fit than the one-factor, χ^2^(3) = 1609.06, *p* < 0.001, the five-factor produced a significantly better fit than either the one- or the three-factor, χ^2^(10) = 2988.64, *p* < 0.001 and χ^2^(7) = 1379.58, *p* < 0.001, respectively. Similarly, for females, the five-factor solution was the best-fitting of the three. Again, in this case, the three-factor solution produced a significantly better fit than the one-factor, χ^2^(3) = 1547.41, *p* < 0.001, but the five-factor model fitted significantly better than either the one- or the three-factor solutions, χ^2^(10) = 3126.53, *p* < 0.001 and χ^2^(7) = 1579.12, *p* < 0.001, respectively.

**Figure 1:**
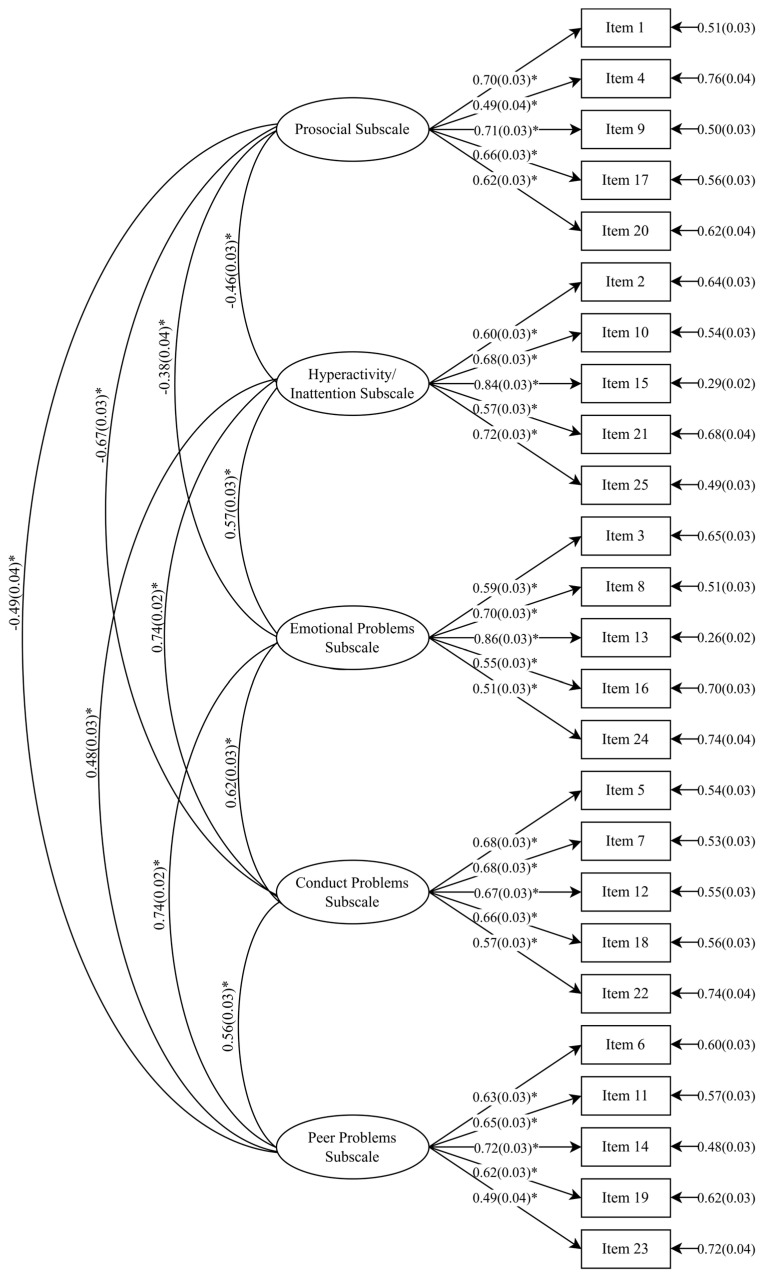
Coefficients from five-factor model of the SDQ for female subsample (* indicates a significant coefficient).

**Figure 2:**
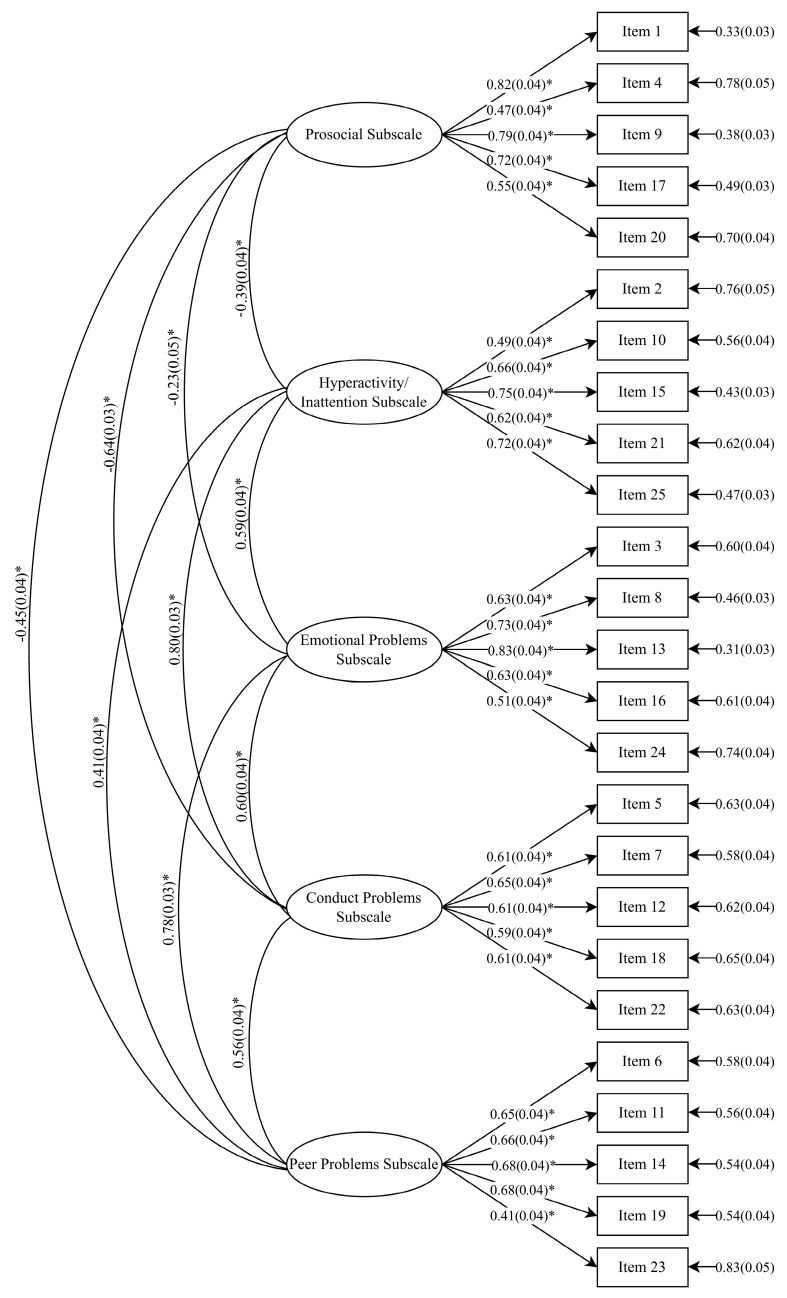
Coefficients from five-factor model of the SDQ for male subsample (* indicates a significant coefficient).

The better fit of the five-factor model was generally further affirmed by other fit indices obtained. These included the Non-Normed Fit Index (NNFI) and the Comparative Fit Index (CFI), both of which assess the degree of fit between proposed and null models (values ≥ 0.90 indicating acceptable model fit); and the Standardized Root Mean Square Residual (SRMR), which tests for discrepancies between the sample covariance matrix residuals and the hypothesized model (values ≤ 0.08 indicating acceptable model fit). Based on these indices, the fit of the five-factor solution was broadly supported. For females, all indices met acceptable cutoffs. For males, all indices met these cutoffs with the exception of the NNFI, which fell marginally short of the 0.90 threshold. The coefficients for paths between each of the items and their respective latent factors are shown in Figure 1 and Figure 2.

### 3.2. Evidence Related to Correlations with Other Variables

To provide a further evaluation of the validity of each of the SDQ subscale scores, these were correlated with external measures that theoretically should correlate with each of the respective subscales in the SDQ. The first subscale tested was the Hyperactivity/Inattention (ADHD) subscale. To evaluate the validity of this subscale, a two-way analysis of variance (ANOVA) was conducted on the total ADHD subscale scores, using answers to the question about whether the participant had ever been diagnosed with ADHD, and gender, as the two independent variables. Only 365 participants provided a definitive answer to this question; a further 27 indicated that they did not know. The remaining participants did not respond to this question. Descriptive statistics for the SDQ ADHD scores are shown in Table 4.

The ANOVA indicated no significant interaction effect for gender by diagnostic category, *F*(2,388) < 1. There was, however, a significant main effect for gender, *F*(1,388) = 5.59, *p* < 0.02, partial η^2^ = 0.01, and also a significant main effect for the diagnostic category, *F*(2,388) = 32.50, *p* < 0.001, partial η^2^ = 0.14. Tukey post-hoc tests, also shown in Table 4, indicated that while the difference between the ‘Yes’ and ‘No’ groups was significant, as expected, reflecting a higher mean score for the former group, the ‘Unsure’ group also differed significantly from the ‘No’ group and did not differ significantly from the ‘Yes’ group. It is possible that those who responded to the question and indicated that they were ‘unsure’ had been referred for ADHD testing, though they were not sure of the outcome of this evaluation. To have been evaluated for the condition, many such participants are likely to have been exhibiting relevant symptoms of ADHD, although they themselves may not have been aware of whether they went on to receive a formal diagnosis.

To evaluate the validity of the Prosocial and Peer Problems subscales of the SDQ, the PALs friendship-related loneliness and isolation subscales, along with the CHKS measure of school belongingness, were used. Given that the SDQ Prosocial subscale is designed to measure participants’ ability to relate well with peers, favoring actions that benefit the individuals with whom they live, it was anticipated that this would correlate positively with both the PALs friendship and the CHKS school belongingness measures, but negatively with the PALs isolation subscale. The opposite patterns of correlation were expected for the SDQ Peer Problems subscale, which is designed to measure difficulties encountered in relating to peers, such as tending to play alone. To assess these relationships, bivariate (Pearson) correlations were performed. The results are shown in Table 5.

As indicated, the pattern of relationships obtained was aligned with the theoretical content of the measures. Specifically, while SDQ Prosocial scores were significantly and positively related to PALs Friendship and CHKS school belongingness scores, they were significantly and negatively related to PALs Isolation scores. The reverse pattern of scores was evident for scores on the SDQ Peer Problems subscale, which were significantly and negatively related to PALs Friendship and CHKS school belongingness scores, and significantly and positively related to PALs Isolation scores.

To evaluate the validity of the SDQ Emotional Symptoms and Conduct Problems subscales, scores from these subscales were correlated with those from the Bullying Victim and Perpetrator measures used, and also those from the CDI-2. Given that the SDQ Emotional Symptoms subscale is intended to measure symptoms of emotional difficulties (e.g., “I am often unhappy, depressed or tearful”), it was anticipated that this would correlate positively with CDI-2 scores and scores on the Bullying Victim measure. As the SDQ Conduct Problems subscale is intended to measure symptoms of misconduct (e.g., “I fight a lot. I can make other people do what I want”), it was anticipated that this would correlate positively with scores from the Bullying Perpetrator measure. The Pearson bivariate correlations obtained are shown in Table 6.

While the anticipated correlations were obtained in this analysis, the results also suggested that scores on the SDQ Emotional Symptoms and Conduct Problems subscales were significantly and positively correlated, as was the correlation between the SDQ Emotional Symptoms scores and the Bullying Perpetrator scores, though the latter correlation was clearly lower than for the Bullying Victim scores. Furthermore, somewhat surprisingly, there was a significant and positive correlation between SDQ Conduct Problems scores and both the Bullying Victim and the CDI-2 scores. This suggests that while conduct problems as measured by the SDQ did identify participants who acted as a perpetrator in bullying incidents (as predicted), participants who were higher on SDQ Conduct Problems were also more likely to report being victims of bullying and to experience depressive symptoms.

## 4. Discussion

The results of this study provide strong support for the validity of the SDQ in terms of its internal structure and relationships with other variables. First, in terms of the internal structure of the SDQ, the best-fitting model of score patterns amongst the items was the original five-factor solution, both for males and for females. Neither the one-factor model nor the alternative three-factor model produced an adequate fit to the data. Thus, in the Western Australian school context, the original proposed five-factor structure for the Youth Self-Report version of the SDQ was the most tenable. While the NNFI for males did fall marginally short of the threshold for a good fit, all other indices met threshold levels for a good fit, supporting the validity of the SDQ in terms of its internal structure.

The results indicated a better fit of the original theoretical model underlying the SDQ than many previous studies on the Youth Self-Report version, which indicated a poor or modest overall fit of the model to the data. While a few of these were conducted in European countries, including England [25], Northern Ireland [34], and the Netherlands [31], one [32] was conducted in Australia. In other studies, modifications such as cross-loadings and correlated error terms [6,21,36,37] or the addition of factors/removal of items have been necessary to achieve adequate fit [28,33,40].

Of the previous studies that did report an adequate fit of the five-factor model to the data, some results suggested the need for cautious inferences about overall support for the model. For example, based on their CFAs, De Vries et al. [12] reported significant item loadings (>0.40) for the Emotional Symptoms and Prosocial Behavior subscales, but not for all relevant items on the other three domains. Similarly, Ruchkin et al. [38] found that while the fit of the five-factor model was sound, the factor loadings were low. Of the studies reviewed, only Muris et al. [39] and Thompson et al. [35] reported unqualified support for the five-factor model of the SDQ. Consequently, the present study proffers stronger support for the internal structure of the SDQ than many previous studies, based on the original five-factor model.

Correlations in this study between the SDQ scores and those from other instruments that test theoretically related concepts also supported the validity of the SDQ. First, scores on the Hyperactivity/Inattention subscale correlated positively with respondents’ self-reported ADHD diagnostic status. Interestingly, those who responded that they were ‘unsure’ to this question also had higher scores on the SDQ Hyperactivity/Inattention (ADHD) subscale than those who affirmed that they had not been diagnosed. It is possible that those who gave this response were aware that they had been tested for ADHD symptoms but may not have been fully apprised of the results of this evaluation (otherwise, an answer of ‘no’ would be expected). For this reason, it is likely these children and adolescents did exhibit some symptoms associated with hyperactivity or inattention, even if they had not been formally diagnosed.

For the Prosocial and Peer Problems subscales of the SDQ, as well as the Emotional Symptoms and Conduct Problems subscales, all relationships that emerged aligned with the theoretical framework underpinning the SDQ. First, scores on the Prosocial Behavior and Peer Problems subscales correlated positively and negatively with the PALs Friendship subscale and CHKS belongingness scores, respectively, while the opposite pattern of relationships was evident for the PALs Isolation subscale scores. The Emotional Symptoms and Conduct Problems subscales were correlated positively with the Bullying Victim, Bullying Perpetrator, and CDI-2 Total scores, which aligns with previous research indicating that children and adolescents with emotional and conduct problems tend to exhibit higher levels of emotional distress [48,49] and participate in bullying more frequently than those without such difficulties [50,51,52]. Also aligned with the theoretical framework for the SDQ was the finding that correlations between scores on the Emotional Symptoms subscale were lower than for scores on the Conduct Problems subscale, though the former relationships were still significant and positive.

## 5. Conclusions

The results of the present study suggest that the Youth Self-Report version of the SDQ may provide a valid, practical, and cost-effective means by which to screen for emotional and behavioral difficulties in Western Australian children and adolescents. Future research could be conducted to compare scores obtained across the different SDQ forms on the same children and adolescents to provide a 360-degree evaluation across youth, parents, and teachers. Subsequent evaluations could also explore ways in which the results of SDQ screening can be deployed to provide additional support for students in need on an ongoing basis. Given that the difficulties assessed by the SDQ in young people typically persist into adulthood [53], such problems not only increase the levels of distress encountered by these individuals but also the long-term financial burden of mental health difficulties to society. This underscores the importance of regular screening using valid tools so that the complex array of challenges that confront adolescents can be addressed in a timely way.

Despite the strengths of this current research, there are limitations that must be acknowledged. Full diagnostic information was not available for all our samples and, as such, there may be issues pertaining to comorbidity, which is the rule rather than the exception with ADHD. Our data are based on adolescent’s self-report and, as such, may be subject to bias and poor recall. Nevertheless, the self-report nature is also a strength. Obtaining insight into the subjective dispositions of young people can only come from themselves. Third parties such as parents and teachers have great difficulty perceiving the internal world and internalizing experiences of their children [54]. Finally, the sample in this research was from Western Australia and therefore the findings may not be generalizable to other cohorts of adolescents. Future research could be conducted to compare scores obtained across the different SDQ forms on the same children and adolescents to provide a 360-degree evaluation across youth, parents and teachers. Subsequent evaluations could also explore the ways in which the results of SDQ screening can be deployed to provide additional support for students in need.

## Figures and Tables

**Table 1:** Versions of the SDQ.

Abbreviation	SDQ Measure
PC1	Parent Report Measure for Children aged 4–10; Baseline version
PC2	Parent Report Measure for Children and Adolescents aged 4–10; Follow up version
PY1	Parent Report Measure for Youth aged 11–17, Baseline version
PY2	Parent Report Measure for Youth aged 11–17; Follow up version
YR1	Youth Self-Report measure (11–17); Baseline version
YR2	Youth Self-Report measure (11–17); Follow up version

**Table 3:** Fit statistics for three models for males and females.

Subgroup	Model	Normal Theory χ^2^	*df*	NNFI	CFI	SRMR
Male	One-factor	4808.50	275	0.78	0.80	0.11
	Three-factor	3199.44	272	0.84	0.85	0.11
	Five-factor	1819.86	265	0.89	0.90	0.08
Female	One-factor	5357.92	275	0.81	0.83	0.10
	Three-factor	3810.51	272	0.86	0.87	0.09
	Five-factor	2231.39	265	0.90	0.91	0.07

**Table 4:** Descriptive statistics for SDQ ADHD scores across diagnostic groups.

ADHD Diagnosis	*n*	*M* (*SD*)	Tukey Post-Hoc Tests			
			Comp.	Diff.	*SE*_Diff_	*p*
No	304	1.84 (0.47)	Yes	−0.45 *	0.06	<0.001
			Unsure	−0.42 *	0.09	<0.001
Yes	61	2.29 (0.35)	No	0.45 *	0.06	<0.001
			Unsure	0.02	0.10	0.975
Unsure	27	2.27 (0.39)	No	0.42 *	0.09	<0.001
			Yes	−0.02	0.10	0.975

* Significant at 0.05 level.

**Table 5:** Descriptives and correlations for SDQ Prosocial and Peer Relations subscales.

Subscale	*n*	*M* (*SD*)	Correlations				
			1	2	3	4	5
1. PALS—Friendships	1487	27.23 (6.68)	--	−0.69 **	0.59 **	0.33 **	−0.62 **
2. PALS—Isolation	1486	11.35 (5.49)		--	−0.51 **	−0.22 **	0.58 **
3. CHKS Belonging	1480	12.08 (2.75)			--	0.40 **	−0.56 **
4. SDQ—Prosocial	1489	2.54 (0.37)				--	−0.29 **
5. SDQ—Peer Problems	1489	1.48 (1.98)					--

** Correlation significant at the 0.01 level.

**Table 6:** Descriptives and correlations for SDQ Emotional Problems and Conduct Problems subscales.

Subscale	*n*	*M* (*SD*)	Correlations				
			1	2	3	4	5
1. SDQ—Emotional Problems	1489	1.81 (0.52)	--	0.34 **	0.33 **	0.13 **	0.68 **
2. SDQ—Conduct Problems	1489	1.48 (0.40)		--	0.37 **	0.40 **	0.52 **
3. Bullying Victim	1466	6.66 (3.10)			--	0.51 **	0.41 **
4. Bullying Perpetrator	1468	5.03 (2.00)				--	0.26 **
5. CDI-2 Depression	1489	6.30 (4.79)					--

** Correlation significant at the 0.01 level.

## Data Availability

The data presented in this study are available upon request from the corresponding author. These are not publicly available given ethical assurances issued to participants in the study.

## References

[1] Lawrence D., Johnson S., Hafekost J., Boterhoven De Haan K., Sawyer M., Ainley J., Zubrick S.R. (2015). The Mental Health of Children and Adolescents: Report on the Second Australian Child and Adolescent Survey of Mental Health and Wellbeing [Young Minds Matter].

[2] NHS Digital (2022). Mental Health of Children and Young People in England 2022: Wave 3 Follow up to the 2017 Survey.

[3] Aebi M., Giger J., Plattner B., Metzke C.W., Steinhausen H.C. (2014). Problem coping skills, psychosocial adversities and mental health problems in children and adolescents as predictors of criminal outcomes in young adulthood. Eur. Child Adolesc. Psychiatry.

[4] Currie J., Stabile M. (2006). Child mental health and human capital accumulation: The case of ADHD. J. Health Econ..

[5] Dalsgaard S., Mortensen P.B., Frydenberg M., Thomsen P.H. (2013). Long-term criminal outcome of children with attention deficit hyperactivity disorder. Crim. Behav. Ment. Health.

[6] Ortuño-Sierra J., Fonseca-Pedrero E., Aritio-Solana R., Velasco A.M., de Luis E.C., Schumann G., Cattrell A., Flor H., Nees F., Banaschewski T. (2015). New evidence of factor structure and measurement invariance of the SDQ across five European nations. Eur. Child Adolesc. Psychiatry.

[7] Colizzi M., Lasalvia A., Ruggeri M. (2020). Prevention and early intervention in youth mental health: Is it time for a multidisciplinary and trans-diagnostic model for care?. Int. J. Ment. Health Syst..

[8] National Mental Health Commission (2019). Monitoring Mental Health and Suicide Prevention Reform—National Report 2019.

[9] Graybill E., Salmon A., Barger B., Roach A.T. (2022). Examining the predictive utility of the self-report Strengths and Difficulties Questionnaire with middle school students. J. Ment. Health.

[10] Hoofs H., Jansen N.W.H., Mohren D.C.L., Jansen M.W.J., Kant I.J. (2015). The context dependency of the self-report version of the Strength and Difficulties Questionnaire (SDQ): A cross-sectional study between two administration settings. PLoS ONE.

[11] He J.-P., Burstein M., Schmitz A., Merikangas K.R. (2013). The Strengths and Difficulties Questionnaire (SDQ): The factor structure and scale validation in U.S. adolescents. J. Abnorm. Child Psychol..

[12] De Vries P.J., Davids E.L., Mathews C., Aarø L.E. (2018). Measuring adolescent mental health around the globe: Psychometric properties of the self-report Strengths and Difficulties Questionnaire in South Africa, and comparison with UK, Australian and Chinese data. Epidemiol. Psychiatr. Sci..

[13] Rothenberger A., Woerner W. (2004). Editorial: Strengths and Difficulties Questionnaire (SDQ)—Evaluations and applications. Eur. Child Adolesc. Psychiatry.

[14] Goodman R. (1997). The Strengths and Difficulties Questionnaire: A research note. J. Child Psychol. Psychiatry.

[15] American Psychiatric Association (1994). Diagnostic and Statistical Manual of Mental Disorders.

[16] American Psychiatric Association (2013). Diagnostic and Statistical Manual of Mental Disorders.

[17] Houghton S., Kyron M., Hunter S.C., Lawrence D., Hattie J., Carroll A., Zadow C. (2022). Adolescents’ longitudinal trajectories of mental health and loneliness: The impact of COVID-19 school closures. J. Adolesc..

[18] Goodman R. (2001). Psychometric Properties of the Strengths and Difficulties Questionnaire. J. Am. Acad. Child Adolesc. Psychiatry.

[19] Hawes D.J., Dadds M.R. (2004). Australian data and psychometric properties of the Strengths and Difficulties Questionnaire. Aust. N. Z. J. Psychiatry.

[20] Hoosen N., Davids E.L., de Vries P.J., Shung-King M. (2018). The Strengths and Difficulties Questionnaire (SDQ) in Africa: A scoping review of its application and validation. Child Adolesc. Psychiatry Ment. Health.

[21] Karlsson P., Larm P., Svensson J., Raninen J. (2022). The factor structure of the Strength [sic] and Difficulties Questionnaire in a national sample of Swedish adolescents: Comparing 3 and 5-factor models. PLoS ONE.

[22] American Educational Research Association (2014). American Psychological Association, and National Council on Measurement in Education. Standards for Educational and Psychological Testing.

[23] Borsboom D., Mellenbergh G.J., van Heerden J. (2004). The Concept of Validity. Psychol. Rev..

[24] Mehrens W.A. (1997). The consequences of consequential validity. Educ. Meas. Issues Pract..

[25] Black L., Mansfield R., Panayiotou M. (2021). Age appropriateness of the self-report Strengths and Difficulties Questionnaire. Assessment.

[26] Jensen S.A., Fabiano G.A., Lopez-Williams A., Chacko A. (2006). The reading grade level of common measures in child and adolescent clinical psychology. Psychol. Assess..

[27] Patalay P., Hayes D., Wolpert M. (2018). Assessing the readability of the self-reported Strengths and Difficulties Questionnaire. BJPsych Open.

[28] Ellis K., Jones F.W., Mallett J. (2014). Differences in the factor structure of the Strengths and Difficulties Questionnaire in Northern Irish children. Peace Confl. J. Peace Psychol..

[29] Van Widenfelt B.M., Goedhart A.W., Treffers P.D., Goodman R. (2003). Dutch version of the Strengths and Difficulties Questionnaire (SDQ). Eur. Child Adolesc. Psychiatry.

[30] Vogels A.G.C., Siebelink B.M., Theunissen M.H.C., Wolff M.S., Reijneveld S.A. (2011). Vergelijking van de KIVPA en de SDQ als signaleringsinstrument voor problemen bij adolescenten in de jeugdgezondheidszorg. Comparison of the KIVPA and SDQ as Used for Screening Among Adolescents in Youth Healthcare.

[31] Vugteveen J., de Bildt A., Theunissen M., Reijneveld S.A., Timmerman M. (2021). Validity aspects of the Strengths and Difficulties Questionnaire (SDQ) adolescent self-report and parent-report versions among Dutch adolescents. Assessment.

[32] Mellor D., Stokes M. (2007). The factor structure of the Strengths and Difficulties Questionnaire. Eur. J. Psychol. Assess..

[33] Duinhof E.L., Lek K.M., de Looze M.E., Cosma A., Mazur J., Gobina I., Wüstner A., Vollebergh W.A.M., Stevens G.W.J.M. (2020). Revising the self-report strengths and difficulties questionnaire for cross-country comparisons of adolescent mental health problems: The SDQ-R. Epidemiol. Psychiatr. Sci..

[34] Percy A., McCrystal P., Higgins K. (2008). Confirmatory factor analysis of the adolescent self-report Strengths and Difficulties Questionnaire. Eur. J. Psychol. Assess..

[35] Thompson J.M.D., Slykerman R.F., Wall C.R., Murphy R., Mitchell E.A., Waldie K.E. (2021). Factor structure of the SDQ and longitudinal associations from pre-school to pre-teen in New Zealand. PLoS ONE.

[36] Bøe T., Hysing M., Skogen J.C., Breivik K. (2016). The Strengths and Difficulties Questionnaire (SDQ): Factor structure and gender equivalence in Norwegian adolescents. PLoS ONE.

[37] Van Roy B., Veenstra M., Clench-Aas J. (2008). Construct validity of the five-factor Strengths and Difficulties Questionnaire (SDQ) in pre-, early, and late adolescence. J. Child Psychol. Psychiatry.

[38] Ruchkin V., Koposov R., Schwab-Stone M. (2007). The Strength and Difficulties Questionnaire: Scale validation with Russian adolescents. J. Clin. Psychol..

[39] Muris P., Meesters C., van den Berg F. (2023). The Strengths and Difficulties Questionnaire (SDQ): Further evidence for its reliability and validity in a community sample of Dutch children and adolescents. Eur. Child Adolesc. Psychiatry.

[40] Skoczeń I., Rogoza R., Maćkiewicz M., Najderska M., Cieciuch J. (2018). Investigating the structural model of the Strengths and Difficulties Questionnaire. Eur. J. Psychol. Assess..

[41] Houghton S., Hattie J., Wood L., Carroll A., Martin K., Tan C. (2014). Conceptualising loneliness in adolescents: Development and validation of a self-report instrument. Child Psychiatry Hum. Dev..

[42] Houghton S., Hattie J., Carroll A., Wood L., Baffour B. (2016). It hurts to be lonely! Loneliness and positive mental wellbeing in Australian rural and urban adolescents. J. Psychol. Couns. Sch..

[43] Houghton S., Lawrence D., Hunter S.C., Zadow C., Kyron M., Paterson R., Carroll A., Christie R., Brandtman M. (2020). Loneliness accounts for the association between diagnosed Attention Deficit-Hyperactivity Disorder and symptoms of depression among adolescents. J. Psychopathol. Behav. Assess..

[44] Resnick M.D., Bearman P.S., Blum R.W., Bauman K.E., Harris K.M., Jones J., Tabor J., Beuhring T., Sieving R.E., Shew M. (1997). Protecting adolescents from harm: Findings from the National Longitudinal Study on Adolescent Health. J. Am. Med. Assoc..

[45] Mahecha J., Hanson T. (2020). Measurement Structure of the California School Climate, Health, and Learning Surveys: Student, Staff, and Parent Surveys.

[46] Masip A.F., Amador-Campos J.A., Gómez-Benito J., Gándara V. (2010). del Barrio. Psychometric properties of the Children’s Depression Inventory in community and clinical sample. Span. J. Psychol..

[47] Goodman A., Lamping D.L., Ploubidis G.B. (2010). When to use broader internalizing and externalizing subscales instead of the hypothesised five subscales on the Strengths and Difficulties Questionnaire (SDQ): Data from British parents, teachers and children. J. Abnorm. Child Psychol..

[48] Factor P.I., Rosen P.J., Reyes R.A. (2016). The relation of poor emotional awareness and externalizing behavior among children with ADHD. J. Atten. Disord..

[49] Gardner D.M., Gerdes A.C. (2015). A review of peer relationships and friendships in youth with ADHD. J. Atten. Disord..

[50] Bacchini D., Affuso G., Trotta T. (2008). Temperament, ADHD and peer relations among schoolchildren: The mediating role of school bullying. Aggress. Behav..

[51] Faraone S.V., Banaschewski T., Coghill D., Zheng Y., Biederman J., Wang Y. (2021). The World Federation of ADHD international consensus statement: 208 evidence-based conclusions about the disorder. Neurosci. Biobehav. Rev..

[52] Wiener J., Mak M. (2009). Peer victimization in children with Attention Deficit/Hyperactivity Disorder. Psychol. Sch..

[53] Australian Institute of Health and Welfare (2022). Australia’s Children.

[54] Baldwin J.S., Dadds M.R. (2007). Reliability and validity of parent and child versions of the multidimensional anxiety scale for children in community samples. J. Am. Acad. Child Adolesc. Psychiatry.

